# Hierarchical Branched Mesoporous TiO_2_–SnO_2_ Nanocomposites with Well‐Defined n–n Heterojunctions for Highly Efficient Ethanol Sensing

**DOI:** 10.1002/advs.201902008

**Published:** 2019-10-24

**Authors:** Tao Zhao, Pengpeng Qiu, Yuchi Fan, Jianping Yang, Wan Jiang, Lianjun Wang, Yonghui Deng, Wei Luo

**Affiliations:** ^1^ State Key Laboratory for Modification of Chemical Fibers and Polymer Materials College of Materials Science and Engineering Institute of Functional Materials Donghua University Shanghai 201620 China; ^2^ Department of Chemistry State Key Laboratory of Molecular Engineering of Polymers Shanghai Key Laboratory of Molecular Catalysis and Innovative Materials iChEM Fudan University Shanghai 200433 China

**Keywords:** co‐assembly, gas sensing, heterojunctions, hierarchical structures, mesoporous TiO _2_

## Abstract

The direct assembly of functional nanoparticles into a highly crystalline mesoporous semiconductor with oriented configurations is challenging but of significance. Herein, an evaporation induced oriented co‐assembly strategy is reported to incorporate SnO_2_ nanocrystals (NCs) into a 3D branched mesoporous TiO_2_ framework by using poly(ethylene oxide)‐block‐polystyrene (PEO‐*b*‐PS) as the template, SnO_2_ NCs as the direct tin source, and titanium butoxide (TBOT) as the titania precursor. Owing to the combined properties of ultrasmall particle size (3–5 nm), excellent dispersibility and presence of abundant hydroxyl groups, SnO_2_ NCs can easily interact with PEO block of the template through hydrogen bonding and co‐assemble with hydrolyzed TBOT to form a novel hierarchical branched mesoporous structure (SHMT). After calcination, the obtained composites exhibit a unique 3D flower‐like structure, which consists of numerous mesoporous rutile TiO_2_ branches with uniform cylindrical mesopores (≈9 nm). More importantly, the SnO_2_ NCs are homogeneously distributed in the mesoporous TiO_2_ matrix, forming numerous n–n heterojunctions. Due to the unique textual structures, the SHMT‐based gas sensors show excellent gas sensing performance with fast response/recovery dynamics, high sensitivity, and selectivity toward ethanol.

## Graphical abstract

A solvent evaporation induced oriented co‐assembly strategy is developed to directly incorporate SnO_2_ nanocrystals into a hierarchical branched mesoporous TiO_2_ nanostructure (SHMT). Owing to the unique properties such as high specific surface area and numerous TiO_2_–SnO_2_ heterojunctions, the SHMT exhibits an excellent ethanol sensing performance, including high sensitivity, fast response/recovery time, and great selectivity.

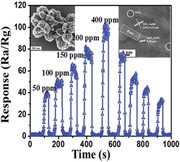

## 1 Introduction

Semiconducting metal oxide (SMO)‐based gas sensors have attracted tremendous attention in the past decade for their wide applications in monitoring air quality, gas leakage, food safety, and medical diagnosis.^1^, ^2^, ^3^, ^4^, ^5^, ^6^, ^7^ Among them, titanium dioxide (TiO_2_) has been extensively studied and represents an exceptional alternative as a gas sensing material due to its excellent properties such as easy production, low‐cost, environmental benignity, and high chemical stability.^8^, ^9^, ^10^ Given that the gas sensing performance of metal oxide semiconductors strongly depends on the surface reactions between the target gases and surface‐chemisorbed oxygen species on the sensing layers,^11^, ^12^, ^13^ rationally design and synthesis of nanostructured TiO_2_ with high surface areas and favorable interfaces is of vital importance.^14^, ^15^, ^16^

As one of excellent candidates, mesoporous materials have inspired increasing attention because of their fascinating features such as large pore sizes, high surface areas, alternative pore shapes, and controllable framework compositions.^17^, ^18^, ^19^, ^20^, ^21^ Compared with their bulk counterparts, mesoporous structure provides more active sites for the interactions between solid framework and gaseous molecules, which are extremely advantageous to the adsorption–desorption and surface reaction involved sensing process. Meanwhile, porous materials with interconnected channels and large pore sizes favor the gas diffusion and thus ensure a fast response and recovery as well as a better sensitivity.^22^, ^23^, ^24^ However, most previously reported mesoporous materials were films and bulk materials with uncontrollable morphology,^22^, ^23^, ^25^, ^26^ which is unfavorable to a rapid adsorption and desorption of gases throughout the entire framework. In contrast, the construction of mesoporous materials with a nano‐sized 3D hierarchical structure may provide a greater pore accessibility, a shorter molecular diffusion length and a larger interface for gas–solid interaction due to their large surface‐to‐volume ratio, which can eventually enhance the gas sensing activity.

Moreover, it has been widely recognized that gas sensors based on a single metal oxide semiconductor are difficult to simultaneously attain an excellent comprehensive performance of sensitivity, selectivity, and stability at an optimum working temperature. Various modification strategies have been reported for improving the gas sensing performance of single SMOs, such as doping with aliovalent ions, loading noble metals, compositing with other semiconductors, and supporting on graphene/carbon nanotubes.^26^, ^27^, ^28^ Among them, the construction of heterostructures between two semiconductors is a promising tactic.^20^, ^29^, ^30^, ^31^, ^32^, ^33^ In particular, stannic oxide (SnO_2_) is one important candidate to couple with TiO_2_ to enhance its sensing performance due to the formation of n–n heterojunction, which can facilitate the electron transfer from TiO_2_ to SnO_2_, promoting the oxygen pre‐adsorption.^34^, ^35^, ^36^ For example, Wang et al. prepared a heterostructure by depositing SnO_2_ nanoparticles on TiO_2_ nanobelts, which showed an improved sensing sensitivity toward ethanol compared with single TiO_2_ and SnO_2_.^34^ Tomer et al. synthesized an Ag‐doped ordered mesoporous SnO_2_–TiO_2_ nanohybrid by a combined nanocasting and wet impregnation process,^37^ and the hybrid exhibited an excellent sensing performance for ethanol. However, in most of the previous works, post‐treatment of porous materials is the very common approach to insert nanoparticles into or onto the framework, which typically requires multiple steps and complicated synthetic conditions.^34^, ^37^, ^38^, ^39^ The co‐assembly of two metal precursors with the template may resolve these problems, but it is not easy to control the reactivity of metal oxide precursors because of their sensitive and different sol–gel chemistry, which may lead to disordered mesoporous structure and undesired phase separation.^27^, ^40^, ^41^, ^42^, ^43^ Therefore, the direct in situ co‐assembly of functional nanoparticles, rather than of a molecular precursor, into a highly ordered mesoporous frameworks with oriented configurations is greatly desired but remains a great challenge.

Herein, we report a robust strategy to synthesize SnO_2_ NCs embedded hierarchical branched mesoporous TiO_2_ (SHMT) composites through an evaporation induced oriented co‐assembly (EIOC) of polymeric micelles of an amphiphilic block copolymer poly(ethylene oxide)‐block‐polystyrene (PEO‐*b*‐PS) with SnO_2_ NCs and titanium butoxide (TBOT). The key in the synthesis is that the SnO_2_ NCs prepared through the polyol method possess a uniform small particle size and abundant hydroxyl groups on its surface, which can easily interact with the PEO segment of the template through hydrogen bonding. This enables us successfully insert SnO_2_ NCs into the mesoporous TiO_2_ pore wall framework through co‐assembly, forming a well‐defined n–n heterojunction. After calcination to remove the template, the SHMT composites exhibit a uniform 3D branched mesoporous structure with a large pore size (9 nm), a high surface area (≈76 m^2^ g^−1^), and a large pore volume (≈0.11 m^3^ g^−1^). Owing to the unique properties, the SHMT‐based gas sensor shows a fast response (≈7 s), quick recovery (≈5 s), and an ultralow detection limit (200 ppb) for ethanol sensing.

## 2 Results and Discussion

**Figure 1:**
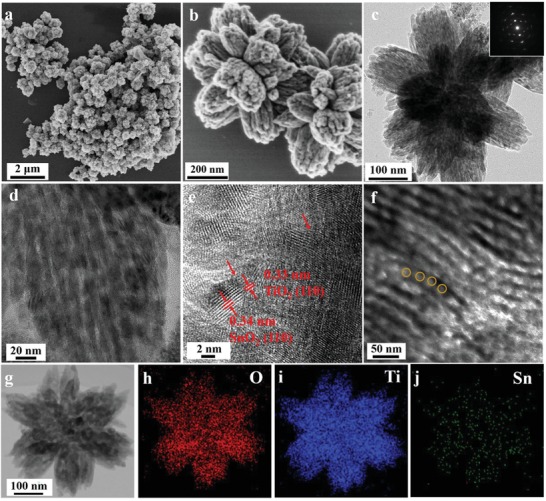
a,b) The FESEM, c,d) TEM, e) HRTEM, and g) STEM images of SHMT composites synthesized via an EIOC approach. f) TEM images of SHMT after ultrathin microtomming. The insert in c) is the SAED pattern; energy dispersive spectrometer elemental mapping of h) O, i) Ti, and j) Sn, respectively.

**Figure 2:**
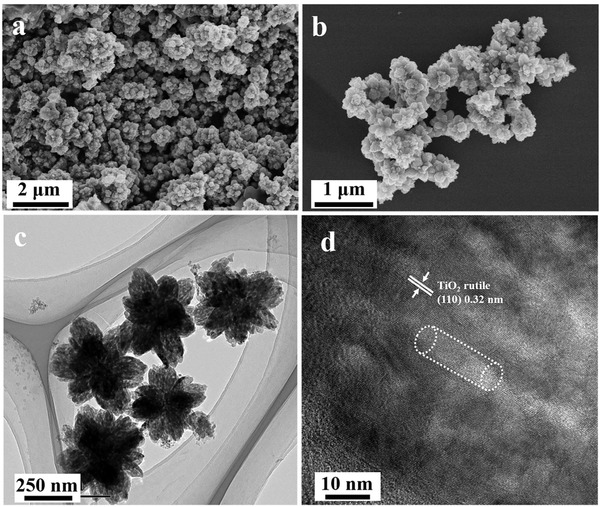
a,b) SEM, c) TEM, and d) HRTEM images of HMT.

At first, SnO_2_ colloidal nanocrystals (NCs) were prepared through a polyol method reported previously.^44^ Transmission electron microscopy (TEM) image (Figure S1a, Supporting Information) shows that the obtained SnO_2_ NCs exhibit a narrow particle size distribution in the range of 3–5 nm (Figure S1d, Supporting Information). Lattice fringes with a *d*‐spacing of ≈0.34 nm can be clearly observed in the high‐resolution TEM (HRTEM) image (Figure S1b, Supporting Information), corresponding well to the (110) plane of the tetragonal cassiterite. In addition, the selected‐area electron diffraction (SAED) pattern shows three clear diffraction rings (Figure S1c, Supporting Information), which can be assigned to (211), (101), (110) planes of cassiterite (JCPDS card no. 41–1445), respectively, implying that the SnO_2_ NCs were well crystallized. Fourier transform infrared spectroscopy results suggest that the surface of SnO_2_ NCs possesses abundant hydroxyl groups (Figure S2, Supporting Information), which could easily associate with PEO segment of the PEO‐*b*‐PS template to co‐assemble with TiO_2_ precursor driven by the hydrogen bonding during the EIOC process. The calcination of the obtained organic–inorganic nanocomposites in N_2_ aims to in situ carbonize PEO‐*b*‐PS template to generate rigid carbonaceous layers on the inner surface of mesopores, which can support the ordered mesoporous structure during crystallization of metal oxides. After further calcination in air for removal of residual carbon, SnO_2_ embedded hierarchical branched mesoporous TiO_2_ (HMT) composites with ordered mesopores and highly crystallized SnO_2_–TiO_2_ frameworks can be obtained. Field‐emission scanning electron microscopy (FE‐SEM) image clearly reveals that the SHMT composites possess a uniform flower‐like structure that was constructed with numerous mesoporous branches subunits growing radially to the center of each particle (**Figure** **1**a). The mesoporous channels can be clearly observed on the surface of each branch unit, resulting in a rough surface. TEM images (Figure 1c, d, and Figure S3, Supporting Information) show that the SHMT composites exhibit a uniform branched mesoporous structure with a particle size of ≈500 nm and the particles consist of numerous mesoporous branches (≈180 nm) with uniform cylindrical mesopores (≈9 nm). Moreover, a series of tilted TEM images ranged from 0° to 30° with an interval degree of 2.5, further prove the flower‐like mesoporous structure is in three dimensions (Figure S4, Supporting Information). The HRTEM image demonstrates that the TiO_2_ in SHMT composites are well crystallized with a *d*‐spacing of ≈0.32 nm, which can be assigned to the (110) plane of the rutile TiO_2_ (Figure S5, Supporting Information). In addition, SnO_2_ NCs with a particle size of ≈4 nm is well embedded in the pore walls (marked in Figure S5, Supporting Information) and tightly attached to TiO_2_ frameworks, resulting in an obvious lattice distortion (Figure 1f), which indicates the formation of a well‐defined heterojunction between the SnO_2_ and TiO_2_. TEM images of the ultramicrotomed sections of SHMT further show the composites contain uniform mesoporous structure (Figure 1f, and Figure S6, Supporting Information). The integrated energy dispersive X‐ray spectroscopy analysis (Figure 1g–j) recorded on a single SHMT particle shows that the elements of Ti, O, and Sn are homogeneously distributed in the entire frameworks. As a comparison, HMT superstructures were also synthesized through the same EIOC process without adding SnO_2_ NCs during the assembly process. Both the SEM and TEM images (**Figure** **2**) reveal that the obtained HMT materials exhibit the similar morphology and mesostructures with SHMT, indicating that the co‐assembly process in this system is robust.

**Figure 3:**
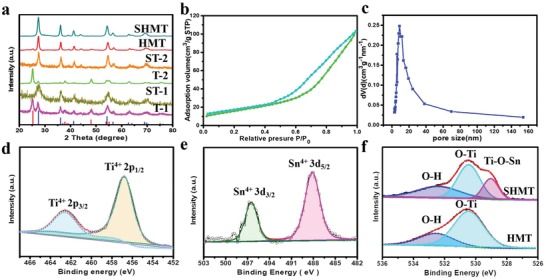
a) XRD patterns of SHMT and HMT at different conditions. T‐1 and ST‐1 represent the as‐made HMT and SHMT before calcination, T‐2 and ST‐2 represent HMT and SHMT after calcination in N_2_; b) nitrogen sorption isotherms, and c) pore size distributions of the SHMT. The pore size distributions are determined by BJH model based on the absorption branches. The high‐resolution XPS spectrum of d) Ti 2p, e) Sn 3d, and f) O 1s spectra.

The presence of SnO_2_ NCs plays an important role in the phase transformation of TiO_2_ during the solvent evaporation and calcination process. X‐ray diffraction (XRD) pattern of the as‐made organic–inorganic hybrid sample of HMT shows a mixed phase of anatase and rutile TiO_2_ owing to the use of too much HCl (concentrated hydrochloric acid).^45^ After calcination at 450 °C in N_2_ and air, the typical peak for anatase TiO_2_ decrease while rutile TiO_2_ increase in HMT, indicating the phase transformation due to the low PH value.^46^ The addition of SnO_2_ NCs during the synthesis could lead to a pure rutile TiO_2_ phase, as shown in the pattern of the as‐made SHMT sample (**Figure** **3**a), which can be explained by the inductive effect of SnO_2_ NCs resulted from the low lattice mismatch between the rutile‐phase SnO_2_ and TiO_2_.^47^ This further confirms a strong interface interaction between TiO_2_ and SnO_2_, which is conducive to the improvement of gas sensing performance. The intensity of the diffraction peak for rutile in SHMT become sharper after calcination, implying an increase of crystallization degree. In addition, no characteristic peaks of SnO_2_ were observed in SHMT sample, which is due to the low content (<5 wt%) of SnO_2_ NCs in the composites. According to the Scherrer equation, the crystal sizes of TiO_2_ in SHMT, and HMT are calculated to be 14.7 and 15.3 nm, respectively, matching well with the HRTEM results. Nitrogen sorption isotherms of the obtained SHMT exhibited type IV curves with a large hysteresis loop in the relative pressure range of 0.45–0.95 (Figure 3b), which are typical for mesoporous materials with a uniform pore size. The pore size distribution derived from the adsorption branch of the isotherms and calculated by Barrett–Joyner–Halenda (BJH) model displays a uniform pore size of ca. 9 nm, originated from the removal of template (Figure 3c). The specific surface area and pore volume of SHMT are calculated to be as high as 76 m^2^ g^−1^ and 0.11 cm^3^ g^−1^, respectively. In addition, the surface area and the pore size of HMT are calculated to be ≈87 m^2^ g^−1^ and 9.3 nm (Figure S7, Supporting Information), respectively. The smaller pore size of SHMT was due to that SnO_2_ NCs was embedded in the pore walls, partially blocking the mesoporous structure. X‐ray photoelectron spectroscopy (XPS) was used to investigate the surface molecular states of the SHMT composites. The survey spectra (Figure S8, Supporting Information) of SHMT shows four well‐resolved peaks of C 1s, Ti 2p, Sn 3d, and O 1s. The C 1s peak at 284.6 eV was attributed to the reference element for calibration. No other peak was detected, suggesting the high purity of as‐prepared sample. The high‐resolution XPS spectra of Ti 2p displays two peaks at 462.6 eV (Ti^4+^ 2p 1/2) and 456.9 eV (Ti^4+^ 2p 3/2), indicating that the Ti element is in the state of Ti^4+^ (Figure 3d).^48^ The peak positions of Sn 3d (Figure 3e) at the binding energies of 495.7 and 487.3 eV can be assigned to the spin orbital splitting of the Sn^4+^ 3d 3/2 and Sn^4+^ 3d 5/2 core level states of tin, respectively, suggesting the standard state of Sn^4+^ in the composites.^49^ The O 1s XPS spectrum of SHMT clearly demonstrates the variation in the chemical states of the oxygen atom (Figure 3f). The spectra can be deconvoluted into three single peaks corresponding to Ti—O (530.6 eV), O—H (532.8 eV), and Ti—O—Sn (529.2 eV) bond, respectively.^35^, ^42^ The appearance of Ti—O—Sn bond suggests a good interface interaction between SnO_2_ and TiO_2_ phases, agreeing well with the HRTEM result. The content of SnO_2_ in SHMT is measured to be ≈4.5 wt% according to XPS results, which is in good agreement with the loading amount during synthesis.

**Figure 4:**
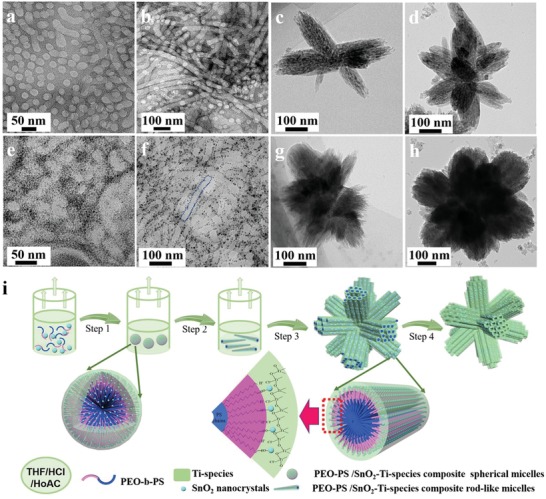
a–d) TEM images of morphology transition diagram of HMT during the experiment. e–h) TEM images of morphology transition diagram of SHMT during the experiment. i) Schematic illustration of the formation process for SnO_2_ NCs embedded HMT through an EIOC approach. Step 1) the formation of composite spherical micelles with PS segments as a core and titania/SnO_2_ NCs‐associated PEO segments as a shell, and Step 2) the spherical micelles fused into 1D nanorods because of the increased concentration of the micelles. Step 3) Composite micelles co‐assemble into a cylindrical bundle with ordered mesostructures, and the bundles array are packed along different direction in order to reduce electrostatic repulsion. Afterwards, 3D hierarchical branched TiO_2_ assembled by cylindrical bundle were generated. Step 4) The template can be carbonized and decomposed with thermal treatment in N_2_ and air, leaving branched TiO_2_ with mesochannels.

To gain insight into the formation mechanism, time‐dependent experiments for both the HMT and SHMT were carried out (**Figure** **4**). At the first stage of the process, the evaporation of THF at 40 °C for 10 h increases the concentration of the mixed solution, inducing the formation of uniform spherical micelles in the solution (Figure S9, Supporting Information). After continuous evaporation of THF for 17 h, the spherical micelles grow larger and the contrast between the PS core and PEO shell becomes clearer, which was due to the continuous crossing‐linking of TiO_2_ frameworks (Figure 4a). When the SnO_2_ NCs was introduced in the solution, the spherical micelles surrounded by plenty of ultrafine nanoparticles were clearly observed (Figure 4e). As evaporation time was prolonged to 20 h, the spherical micelles disappeared and instead, cylindrical micelles were formed (Figure 4b, f). The second stage of evaporation at 80 °C for 2 h could lead to the formation of hierarchical TiO_2_ with a few branches (Figure 4c, g). With the evaporation time prolonged to 6 h, uniform hierarchical branched TiO_2_ particles with numerous branches growing radially to the center of each particle was obtained (Figure 4d, h).

Based on the observation above, an EIOC mechanism was proposed, as illustrated in Figure 4i. With the evaporation of low‐boiling point solvent tetrahydrofuran (THF) which is good solvent for both PEO and PS segments Step 1), the dissolving power of the solvent decreases due to the increased amount of water which is a poor solvent for PS segment but a good one for PEO segment. As a result, the PEO‐*b*‐PS copolymers can form spherical composite micelles with PS core and PEO shell. Meanwhile, the hydrolysis of TBOT leads to the formation of titania oligomers, which could associate with PEO segment via hydrogen bonding. Owing to the presence of abundant hydroxyl groups, SnO_2_ NCs can be easily adsorbed on the PEO shell and encapsulated by the continuous grown TiO_2_ framework. With the continuous evaporation of THF at 40 °C, the spherical micelles tend to contact and fuse along one direction to form 1D nanorods (Figure 4f), resulting in the formation of pod‐like core–shell micelles with PS segments as a core and titania/SnO_2_‐associated PEO segments as a shell Step 2). As the composite micelles become more concentrated with continuous evaporation of THF, they further co‐assemble into bundles with ordered rod‐like micelles arrays driven by the balance of repulsion force of negatively charged rods and the van der Waals attraction force. It causes the bundles to co‐assemble along different direction in order to reduce electrostatic repulsion Step 3), resulting in the formation of SnO_2_ embedded HMT composites. As to the HMT without SnO_2_ as additive, the formation process is quite similar (Figure 4a–d). Furthermore, this method could be extended to synthesize mesoporous TiO_2_ microspheres and mesoporous TiO_2_ bulk via adjusting the evaporation rate of solution (changing the reaction temperature, Figure S10, Supporting Information). For the case of mesoporous bulk TiO_2_, the PEO‐*b*‐PS/Ti‐species composite spherical micelle become concentrated with continuous evaporation of THF at relative high temperature (80 °C), then co‐assemble into ordered mesostructure driven by the balance of repulsion force of negatively charged rods and the van der Waals attraction, as the similar process of previous report.^25^ And the PEO‐*b*‐PS/Ti‐species composite micelles can assemble into sphere structure by using lower evaporation temperature (20 °C) to slowing down THF evaporation ratio, due to the requirement of minimization of interface energy.

**Figure 5:**
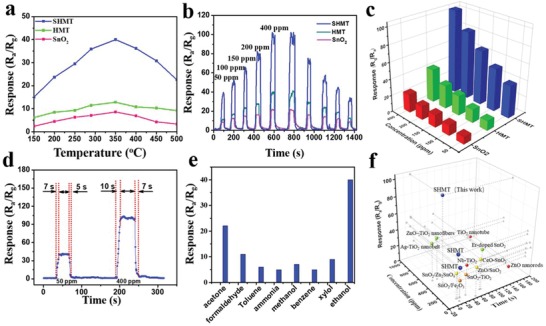
a) Responses of HMT, SnO_2_ NCs, and SHMT‐based sensors toward 50 ppm ethanol at different operating temperatures (150–500 °C). b) Response curves of gas sensors based on SnO_2_ NCs, HMT, and SHMT materials toward different concentration of ethanol (50–400 ppm) at 350 °C, c) responses of the sensors based on SnO_2_ NCs, HMT and SHMT sensors to ethanol vapor under different concentrations (50–400 ppm). d) Dynamic response‐recovery curve of the SHMT to 50 and 400 ppm ethanol. e) Responses of SHMT sensor to various gases at 50 ppm to test the selectivity. f) Comparison of the response and response/recovery time based on SMO sensors with our work (details information of SMO was listed in Table S1 in the Supporting Information).

Owing to the high surface area, large pore size, and well‐defined heterojunction, the obtained SHMT can be an ideal candidate for gas sensing application. The responses of the SnO_2_ NCs, HMT, and SHMT based sensors toward 50 ppm ethanol gas were examined in the temperature range of 150–500 °C (**Figure** **5**a). The responses for all the sensors increase with a rise of operating temperature and attains a maximum value at 350 °C, where after, the responses decrease. The low sensitivity at a lower operating temperature could be explained by the low active energy for the chemisorption of gas species on the surface of materials. While at a higher temperature, gas molecules tend to desorb from surface, thus decreasing the response of the gas sensors.^22^ In addition, SHMT exhibit the maximum response of 40 at 350 °C, which is 3.3 and 4.0 times larger than that of HMT and SnO_2_, respectively. This is because abundant heterojunction present in the SHMT framework is beneficial for the improvement of gas sensing performance.

The continuous dynamic response/recovery curves of the four ethanol vapor sensors based on SnO_2_ NCs, HMT and SHMT were tested at 350 °C (Figure 5b) upon the exposure to ethanol vapor at varied concentrations (50–400 ppm). As the ethanol gas with a larger concentration was injected into the tested chamber, the response of all sensors rapidly increased. As gas out, the responses almost recover to its initial value. The response ratio of the three sensors were summarized in Figure 5c. SHMT‐based sensor displays the best sensing performance, including high response and short response/recovery time (Figure S11, Supporting Information), which can be attributed to the presence of ordered mesopores. Of particular importance, SHMT also showed a rapid response to low concentration of ethanol in the range from 0.2 to 20 ppm (Figure S12, Supporting Information). With the increase of ethanol concentration, the response of SHMT sensor increased dramatically from 4.1 at 0.2 ppm to 40 at 50 ppm, and reached 102 at 400 ppm, exhibiting a much higher sensitivity than the HMT‐ and SnO_2_‐based sensors. This high sensitivity can be credited to the high surface area and n–n heterojunction. The detailed response of SHMT sensor to ethanol gas of different concentrations ranging from 0.2 to 400 ppm is shown in Figure S13 in the Supporting Information. The SHMT‐based sensor shows a fast response (7 s) and quick recovery (5 s) during the process of adsorption and desorption of ethanol gas at a concentration of 50 ppm (Figure 5d). Moreover, it even possesses a fast response and recovery time (10/7 s) toward a high ethanol concentration of 400 ppm. For practical application, the selectivity of the gas sensor is of vital importance. The responses of several interfering gas sources, such as acetone, formaldehyde, toluene, ammonia, methanol, benzene, and xylene, were measured at a constant concentration of 50 ppm at 350 °C (Figure 5e). The response of SHMT‐based sensor to ethanol gas was at least two times larger than other interfering gases, indicating that SHMT possesses a superior selectivity, much better than the HMT sensor. More importantly, the SHMT‐based ethanol sensor shows a much better comprehensive performance than most of previously reported ethanol sensors (Table S1, Supporting Information and Figure 5f).^50^, ^51^, ^52^, ^53^, ^54^, ^55^, ^56^

The excellent comprehensive sensing performance of SHMT‐based ethanol sensors can be ascribed to the unique textual structures of SHMT. On one hand, the hierarchical branched mesoporous structure with a large pore size, high crystalline frameworks, a high surface area provides more active sites and shortens the distance for the diffusion and rapid transfer of gas molecules across its surface, and become the deciding factor for determining the sensitivity and response time of the gas sensor. The 3D interconnected crystalline framework ensures a fast transport of charge carriers from the surface to bulk and sustains an of gas sensing. No obvious reduction of the sensing performance toward 50 ppm ethanol after continuously working for 30 d suggests the excellent long‐cycle stability of the SHMT composites. (Figure S14, Supporting Information). In order to further elucidate the structural effect, the sensing performances of HMT and bulk mesoporous titania (mesoporous titania prepared using EIOC process via adjusting the evaporation ration of solution) toward 100 ppm ethanol were compared (Figure S15 and Table S2, Supporting Information). HMT‐based sensor not only exhibits a higher sensitivity, but also shows a much faster response/recovery time than bulk mesoporous titania, further confirming the superiority of the hierarchical structure built with mesoporous branches. On the other hand, the well‐defined heterojunctions between TiO_2_ matrix and SnO_2_ NCs not only enhance the performance but also improve the selectivity of the sensor. When the two semiconductors come into contact and are subjected to a high temperature treatment, band alignment occurs driven by the equilibration of the Fermi level (Figure S16, Supporting Information).^23^, ^57^ As a result, an n–n type heterojunction is generated at the interface, driving the electron transfer from TiO_2_ to SnO_2_ and thus forming an “accumulation layer” on SnO_2_ rather than a depletion layer. The accumulation layer can be depleted by subsequent oxygen adsorption, further facilitating oxygen pre‐adsorption on the surface of SHMT and increasing the potential energy barrier at the interface, and enhancing the response.^35^, ^57^ The resistance transients of SnO_2_ NCs, HMT, and SHMT toward 200 ppm ethanol gas were measured at their optimizing sensing temperature, respectively (Figure S17, Supporting Information). Obviously, the resistance of SHMT exhibits the largest and fastest decrease trend upon exposing to ethanol, further confirming the advantage of heterojunction between SnO_2_ NCs and TiO_2_ framework. It is worth noting that, the uniform distribution of SnO_2_ NCs in SHMT is very important to sensing performance of the composites. When more SnO_2_ NCs (6 wt%) were introduced in the synthesis, the aggregation of SnO_2_ NCs can occur due to their high surface energy, and it causes an inhomogeneous distribution of SnO_2_ NCs in SHMT. Therefore, the obtained SHMT (6 wt%) exhibits lower sensitivity to ethanol compared with SHMT (4.5 wt%.) (Figure S18, Supporting Information).

## 3 Conclusion

In summary, a facile solvent EIOC has been demonstrated for the synthesis of novel hierarchical branched mesoporous TiO_2_–SnO_2_ semiconducting heterojunctions. During the solvent evaporation process, the amphiphilic PEO‐*b*‐PS copolymers associate with SnO_2_ NCs and TiO_2_ precursor to co‐assemble into mesostructured composite particles which can be converted into mesoporous composites with numerous n–n junctions via calcination. The obtained materials possess a unique flower‐like morphology with a mean diameter of ≈500 nm, which are constructed by several mesoporous rutile branches with a uniform pore size (≈9 nm) and a high specific Brunauer–Emmett–Teller (BET) surface area (76 m^2^ g^−1^). The uniform distribution of SnO_2_ NCs in the pore walls of TiO_2_ forms numerous n–n heterojunctions which are extremely useful for surface catalytic reaction. Owing to the rational combination of a hierarchical mesoporous structure, a high crystallinity, and well‐defined n–n heterojunctions, the SHMT‐based gas sensor shows an excellent sensing performance toward ethanol vapor with a fast response (7 s) and recovery (5 s) dynamics, ultralow limit of detection (200 ppb) and a superior selectivity, which is favorable for development of high‐performance gas sensors. Furthermore, this approach can be used to construct mesoporous TiO_2_ with various morphologies, including the HMT, ordered mesoporous TiO_2_ microspheres and mesoporous TiO_2_ film, which holds a great promise in designing new structural mesoporous materials for various applications. Considering the simplicity of the synthetic strategy and excellent gas sensing performance of the branched nanostructures, it is expected that this co‐assembly process can be extended to develop other hierarchical branched mesoporous semiconductors with different compositions for various applications such as catalysis, gas sensing, energy conversion and storage, etc.

## 4 Experimental Section

*Chemicals*: Monomethyl poly(ethylene oxide) (*M_w_* = 5000 g mol^−1^) (designated as PEO5000) was purchased from Sigma–Aldrich (USA). Copper(I) bromide, styrene (St), pyridine, and Al_2_O_3_ were obtained from Shanghai Chemical Reagent Co. Ltd. N, N, N′, N″, N″‐Pentamethyl diethylenetriamine was provided by Acros. THF, anhydrous ethyl ether, titanium tetrabutoxide(TBOT), acetic acid (AA), HCl (36 wt%) were purchased from Sino‐Pharm Chemical Reagent Co. Ltd. St was purified by passing through an Al_2_O_3_ packed column.

*Synthesis of SHMT*: SnO_2_ NCs was first prepared using a polyol method.^34^ Then, the SnO_2_ embedded HMT superstructure was synthesized through an EIOC approach in an acidic THF/PEO‐*b*‐PS/HCl/AA/ TBOT mixed solution. In a typical synthesis, 0.035 g SnO_2_ NCs, 0.4 g of lab‐made PEO‐*b*‐PS (*M_w_* = 25 600 g mol^−1^, PDI < 1.2), 2.0 g of AA and 2.0 g of concentrated HCl were dissolved in 20 mL of THF. The mixture was stirred vigorously for 30 min to form a clear and transparent solution. Then, 3.0 mL of TBOT was added dropwise under vigorous stirring for 2 h to form a clear red solution. The obtained solution was transferred to a volumetric flask, and left it in a drying oven to evaporate the THF at 40 °C for 20 h, and then at 80 °C for another 8 h to completely remove the solvents. To retain the mesostructure, the obtained milky white precipitate was first pyrolyzed in N_2_ atmosphere at 450 °C for 2 h to obtain a dark grey carbon‐supported SHMT powder. Finally, the white SHMT powder was obtained by further calcination of the carbon‐supported SHMT in air at 450 °C for 1 h to burn out the carbon supports.

*Characterization and Measurements*: TEM images were taken on a JEOL 2100 microscope (Japan) operated at a voltage of 200 kV. FE‐SEM experiments were carried out on a Hitachi model S‐4800 field emission scanning microscope. Wide‐angle XRD patterns were recorded on a Bruker D8 powder X‐ray diffractometer (Germany) with Ni‐filtered Cu Kα radiation (40 kV, 40 mA). Nitrogen sorption isotherms were tested at 77K with a Micromeritics Tristar 3020 analyzer. Prior to the measurements, the samples were degassed under vacuum at 180 °C for at least 6 h. The specific surface areas were calculated based on the adsorption data in the relative pressure range of *P*/*P*_0_ = 0.04–0.2 using the BET method. The pore size distributions derived from the adsorption branches of isotherms were calculated using the BJH model and the total pore volumes (*V*) were estimated according to the adsorbed amount at a relative pressure *P*/*P*_0_ of 0.995.

*Gas Sensing Tests*: The schematic diagram of the ethanol vapor sensor was depicted in Figure S19 in the Supporting Information. The obtained samples were mixed with a calculated amount of water and ground in a mortar to form a paste. Then the paste was coated directly onto the outer surface of ceramic tube and calcinated in air at 300 °C for 2h. A Cr–Ni alloy wire was inserted into the ceramic tube as a heater, and the working temperature could be adjusted by tuning the heating voltage. Finally, the sensor was aged at 290 °C for 48 h to further improve the long‐term stability. Vapor detection was carried out with a WS‐30A gas sensing system (Winsen Electronics Technology Co. Ltd., China) by using a static state gas distribution method. For ethanol detection, an ethanol‐air mixed gas was prepared by injecting a certain volume of liquid ethanol into the test chamber on a heating platform in which the evaporation of ethanol was spread by two small electric fans. The volume of chamber is ≈18 L, and therefore, evaporation of 2.33 µL absolute ethanol results in 50 ppm ethanol vapor. For preparation of 200 ppb ethanol gas, 83.88 µL of liquid ethanol was first evaporated in another 18 L gas chamber to generate 1800 ppm ethanol vapor, then 2.00 mL of the 1800 ppm ethanol vapor was injected to testing chamber (18 L), forming ethanol vapor of 0.2 ppm. In the electric circuit for measuring the gas response (Figure S19b, Supporting Information), a load resistor (*R_L_*) was connected in series with a gas sensor. The circuit voltage (*V_c_*) was set at 5 V, and the output voltage (*V*_out_) was the terminal voltage of the load resistor. The sensitivity (*S*) of the gas sensors is defined as *S = R_a_/R_g_*, where *R_a_* and *R_g_* are the resistance of a sensor in air and tested gas, respectively. The response time is defined as the time required for the variation in conductance to reach 90% of the equilibrium value after a test gas was injected while the recovery time is the time required for the sensor to return to 10% above the original conductance in air after releasing the test gas.

## Conflict of Interest

The authors declare no conflict of interest.

## Supporting information

Supporting Information
