# Supplementary material for: Hierarchical Branched Mesoporous TiO2–SnO2 Nanocomposites with Well‐Defined n–n Heterojunctions for Highly Efficient Ethanol Sensing
Source: Adv Sci (Weinh). 2019 Oct 24;6(24):1902008. doi: 10.1002/advs.201902008 (PMC6918105; doi:10.1002/advs.201902008)
Supplement: Supplementary file 1 — Supporting Information [file ADVS-6-1902008-s001.pdf]

Copyright WILEY-VCH Verlag GmbH & Co. KGaA, 69469 Weinheim, Germany, 2019.

ADVANCED SCIENCE
Open Access

Supporting Information

for *Adv. Sci.,* DOI: 10.1002/advs.201902008

Hierarchical Branched Mesoporous TiO_2–SnO_2 Nanocomposites with Well-Defined n–n Heterojunctions for Highly Efficient Ethanol Sensing

*Tao Zhao, Pengpeng Qiu, Yuchi Fan, Jianping Yang, Wan Jiang, Lianjun Wang, Yonghui Deng, and Wei Luo**

Copyright WILEY-VCH Verlag GmbH & Co. KGaA, 69469 Weinheim, Germany, 2019.

Supporting Information

Hierarchical Branched Mesoporous TiO_2 –SnO_2 Nanocomposites with Well-defined n-n Heterojunctions for Highly Efficient Ethanol Sensing

Tao Zhao, Pengpeng Qiu, Yuchi Fan, Jianping Yang, Wan Jiang, Lianjun Wang, Yonghui Deng,* Wei Luo*

T. Zhao, Dr. P. Qiu, Prof. Y. Fan, Prof. J. Yang, Prof. W. Jiang, Prof. L. Wang, Prof. W. Luo

State Key Laboratory for Modification of Chemical Fibers and Polymer Materials

College of Materials Science and Engineering

Institute of Functional Materials

Donghua University

Shanghai 201620, China

E-mail: wluo@dhu.edu.cn

Prof. Y. Deng

Department of Chemistry

State Key Laboratory of Molecular Engineering of Polymers

Shanghai Key Laboratory of Molecular Catalysis and Innovative Materials

iChEM

Fudan University

Shanghai 200433, China

E-mail: yhdeng@fudan.edu.cn

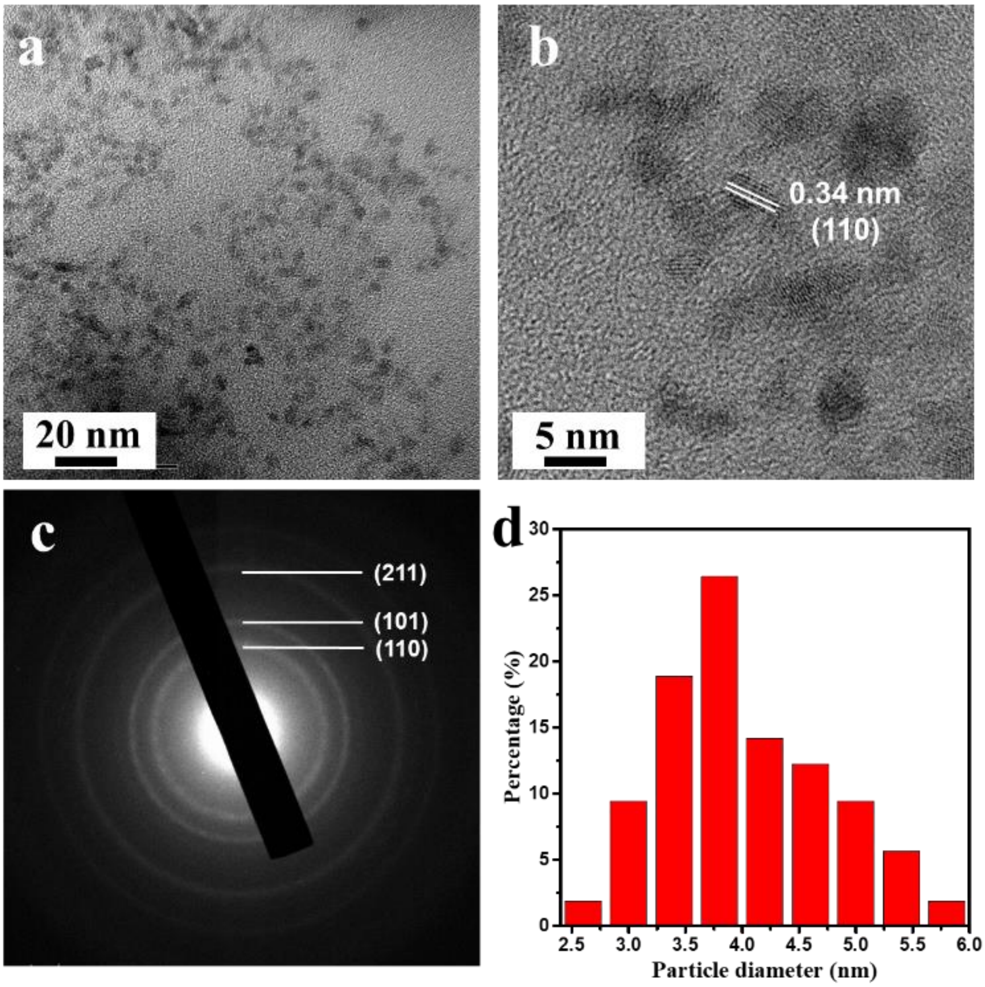

Figure S1. TEM image (a), HRTEM images (b), SAED pattern (c) and particle size distribution curve (d) of SnO_2 NCs.

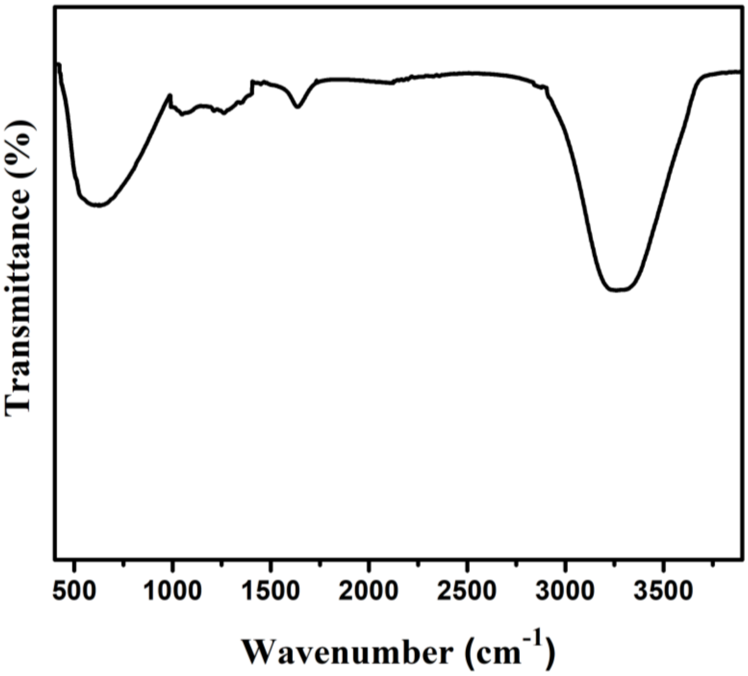

Figure S2. Fourier transform infrared spectroscopy (FTIR) spectrum of SnO_2 NCs.

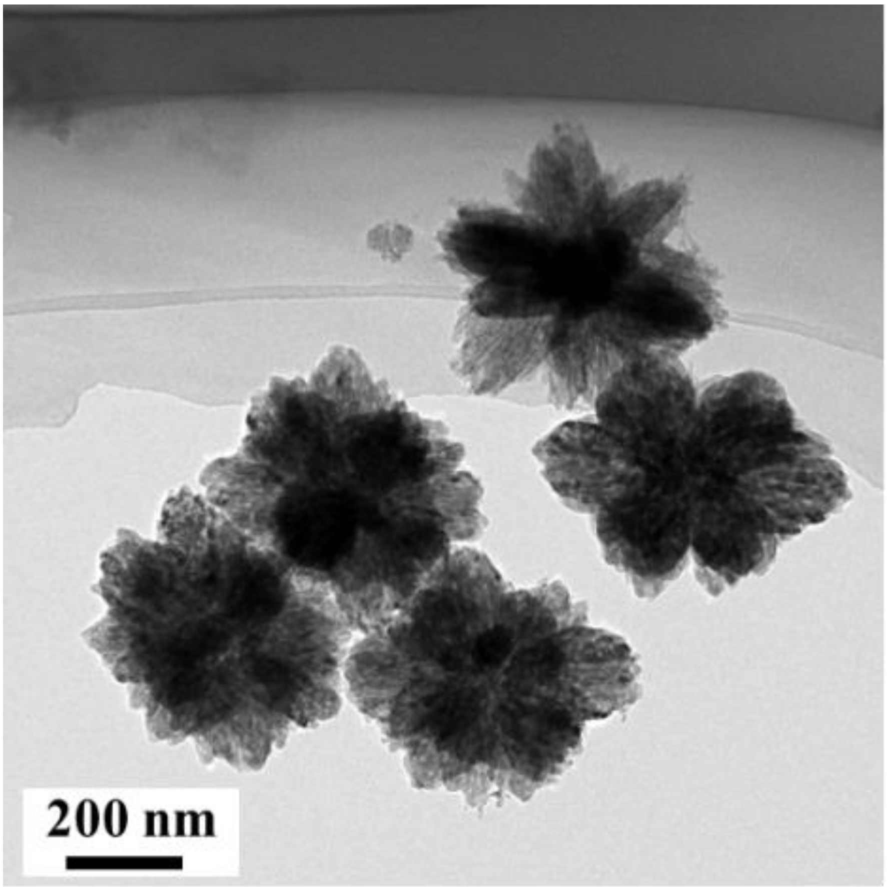

Figure S3. TEM image of SHMT synthesized via an evaporation induced oriented co-assembly (EIOC) approach.

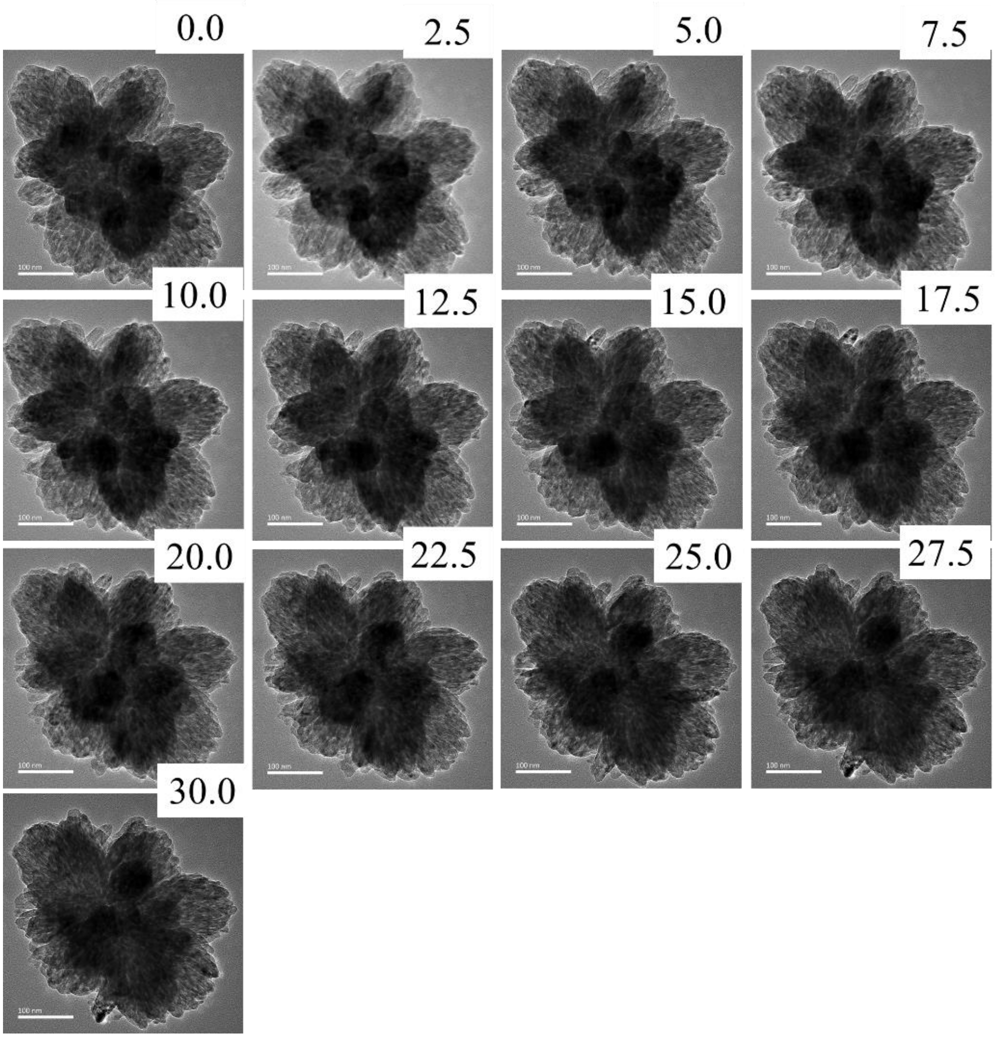

Figure S4. Tilted TEM image of SHMT synthesized *via* an EIOC approach.

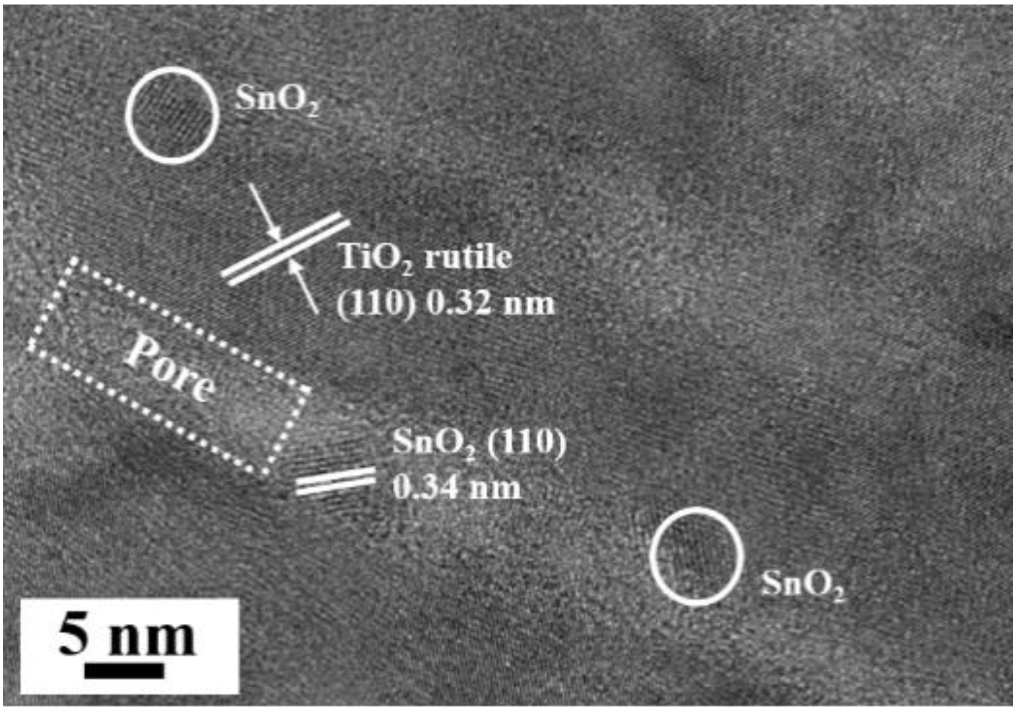

Figure S5. High resolution TEM (HRTEM) image of SHMT.

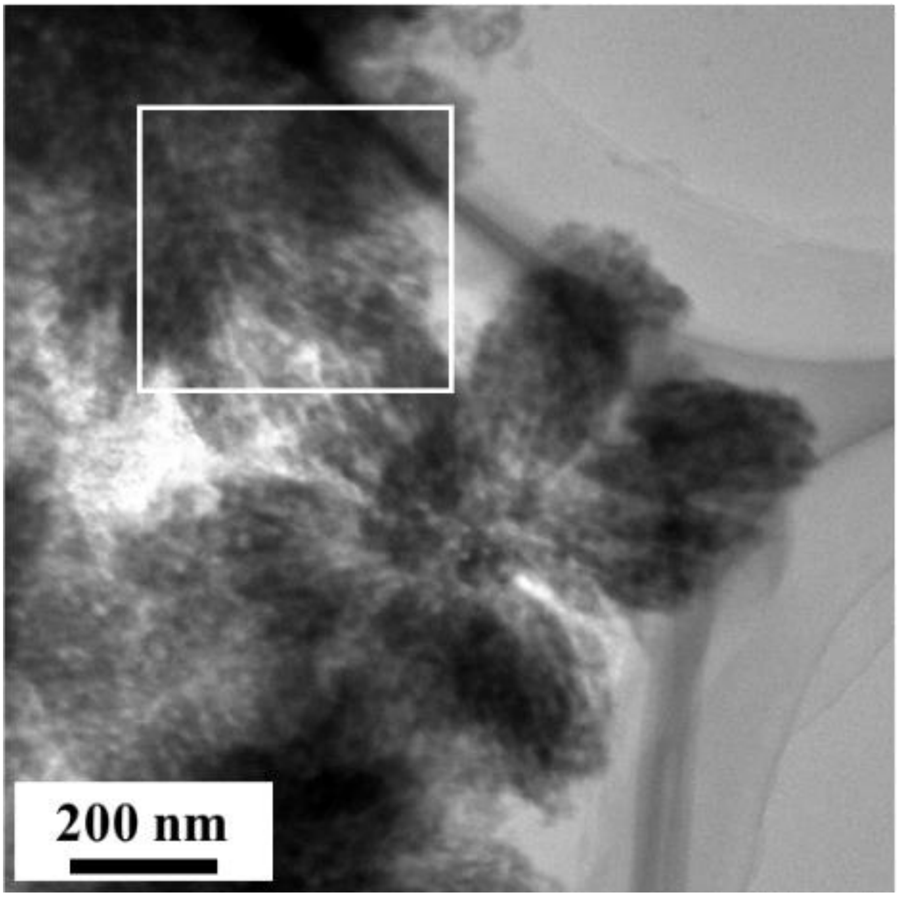

Figure S6. TEM image of ultramicrotomed SHMT, which clearly represents the central symmetric cylindrical pore distribution of the 3D hierarchical branch-like mesoporous titania with an 3D-opened pore structure on the surface.

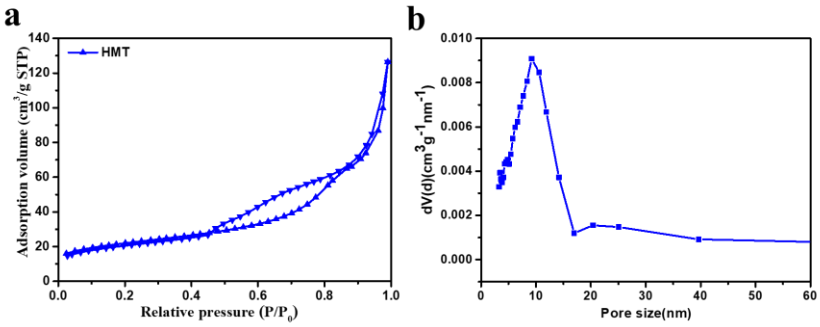

Figure S7. Nitrogen sorption isotherms (a) and pore size distributions (b) of the HMT

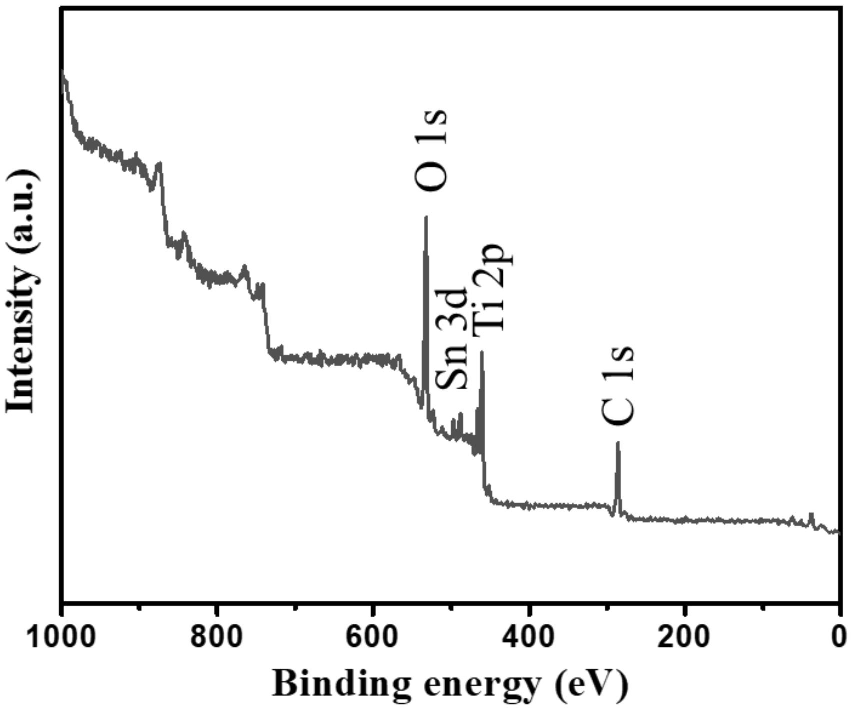

Figure S8. XPS survey spectrum of SHMT.

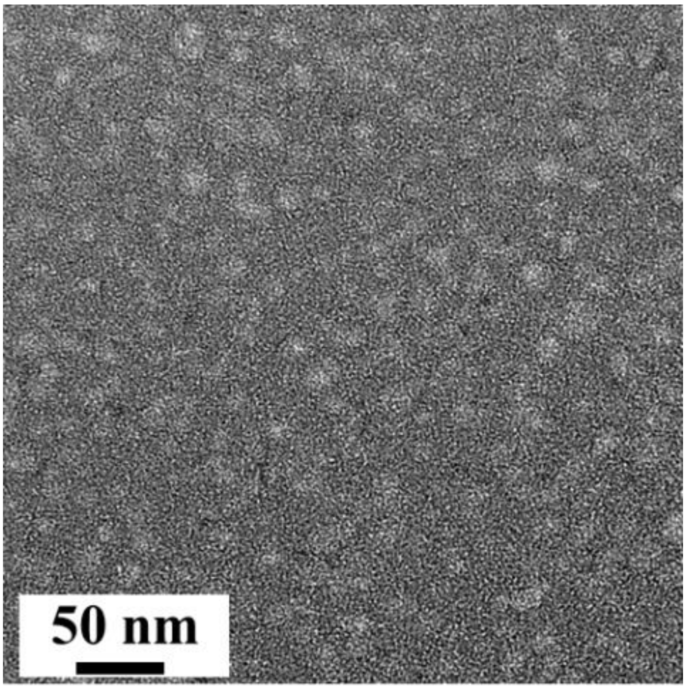

Figure S9. TEM image of the PEO-*b*-PS/titania oligomer composite spherical micelles after evaporation of the solution for 10 h at 40 °C.

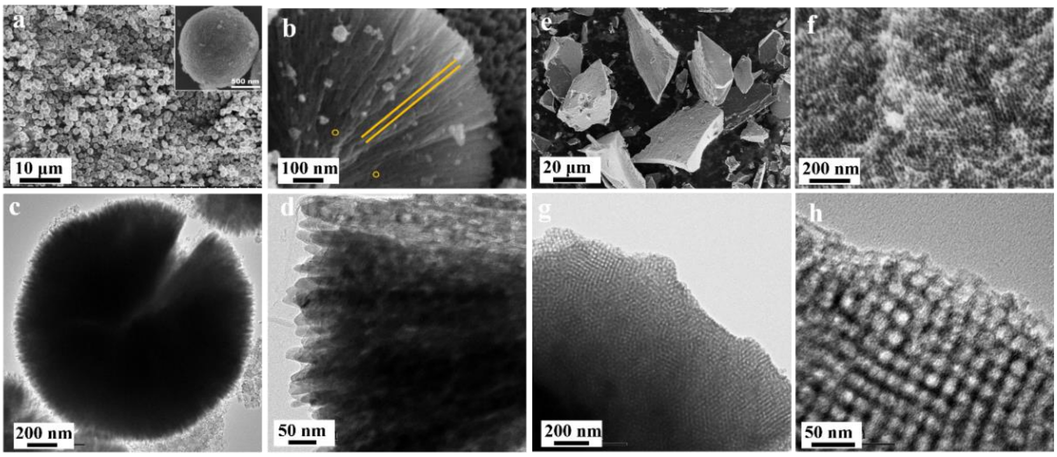

Figure S10. SEM (a) and TEM (c) images of mesoporous TiO_2 spheres synthesized by evaporation induced oriented co-assembly approach *via* slowing down the evaporation ratio of THF. FESEM (b) and TEM (d) images of mesoporous TiO_2 spheres after grinding to observe inside structure. FESEM (e, f) and TEM (g, h) images of mesoporous TiO_2 synthesized via accelerating the evaporation ratio of THF.

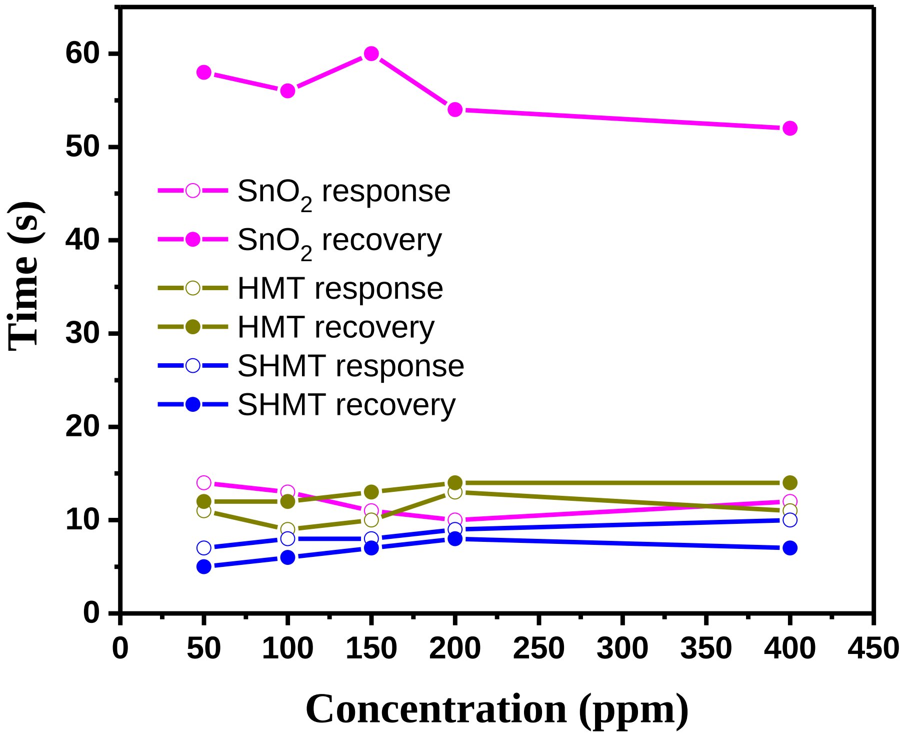

Figure S11. Response and recovery times of the sensor based on SnO_2 NCs, HMT and SHMT at different ethanol concentration.

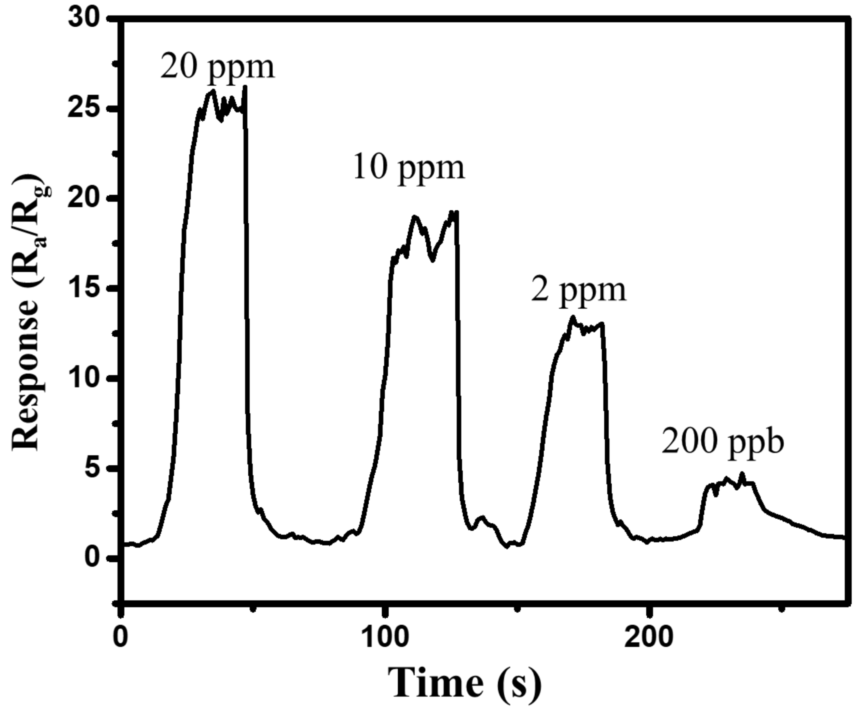

Figure S12. In-situ response-recovery curve of SHMT based vapor sensor toward a low concentration of ethanol (0.2-20 ppm).

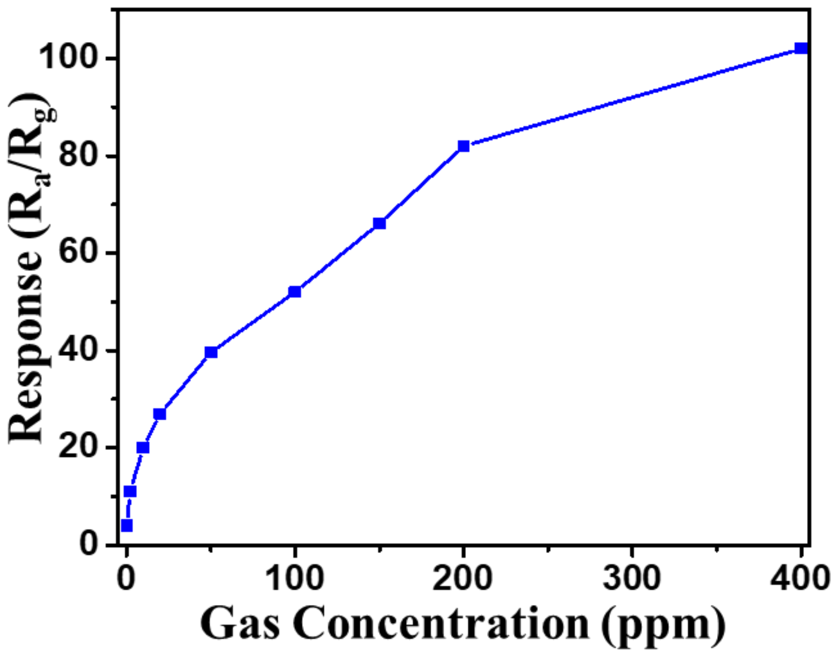

Figure S13. Relationships between ethanol concentration and response of SHMT sensor to ethanol vapors of different concentrations (0.2-400 ppm).

Table S1. Comparison of ethanol sensing performances

Materials and morphology	Concentration (ethanol, ppm)	Sensitivity (R_a/R_g)	Response time/ recovery time(s)	Detection limit	Ref
SnO_2-TiO_2 nanobelt	100	25	>15/>20	10	S1
TiO_2 nanotube array	1000	5	11/170	10	S2
ZnO nanorods	250	2.3	16/120	12.5	S3
Nb-TiO_2 nanorods	400	12	3/79	50	S4
Ag-TiO_2 nanobelt	500	46	1/2	20	S5
ZnO–TiO_2 nanofibers	500	50.6	5/10	20	S6
SnO_2/Zn_2SnO_4 Porous spheres	100	30.5	1/10	0.5	S7
CeO-SnO_2 Hollow Spheres	100	37	2/70	10 ppm	S8
ZnO/SnO_2 Hollow spheres	30	34.8	1/50	0.5 ppm	S9
SnO_2/Fe_2O_3 hollow nanofiber	100	20.4	5/15	2ppm	S10
Er-doped SnO_2	100	48	35/40	20 ppm	S11
SHMT	50	40	7/5 (s)	0.2 ppm	This work

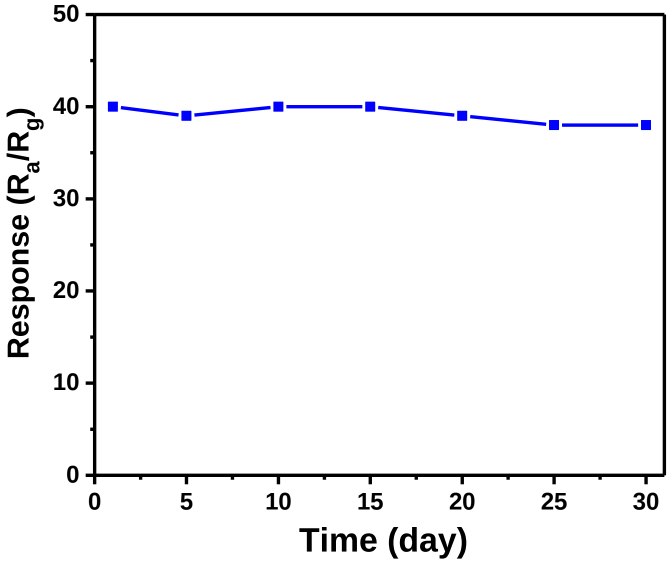

Figure S14. Responses to 50 ppm ethanol for the stability test.

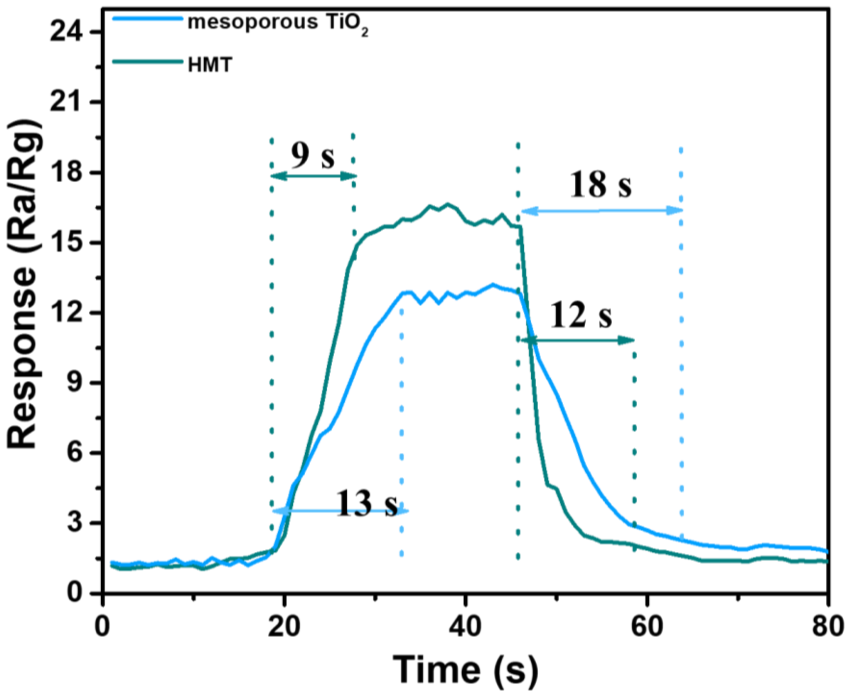

Figure S15. Effect of titanium oxide structure on sensing performance toward 100 ppm ethanol.

Table S2 The results of TiO_2-based sensors toward 100 ppm ethanol

Sample (TiO_2)	Sensitivity	Response time	Recovery time
HMT	16.6	9 s	12 s
Mesoporous film	12.5	13 s	18 s

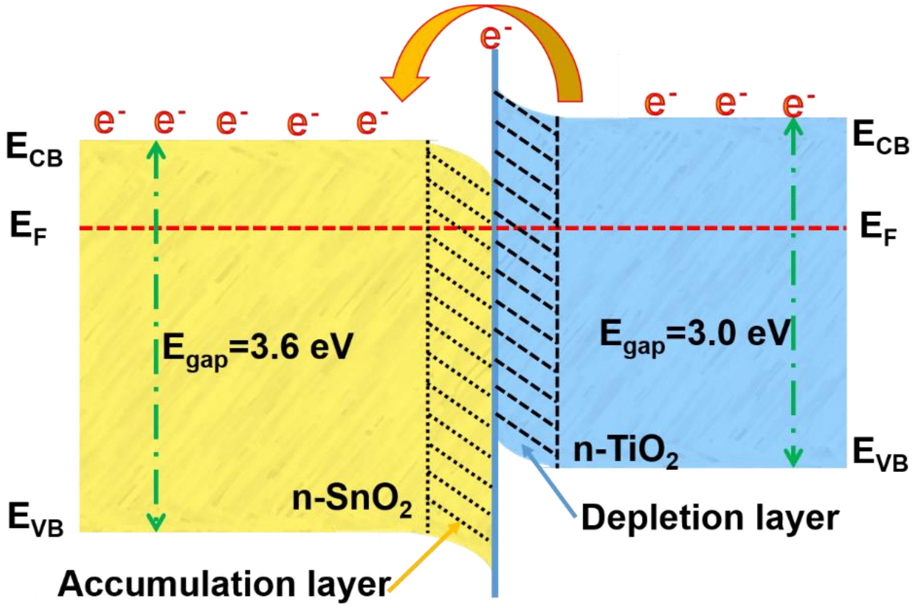

Figure S16. Schematic illustration of the formed TiO_2-SnO_2 n-n heterojunction. E_{CB}: conduction band edge energy, E_{VB}: valence band edge energy, E_{gap}: band gap energy, E_F: Fermi energy.

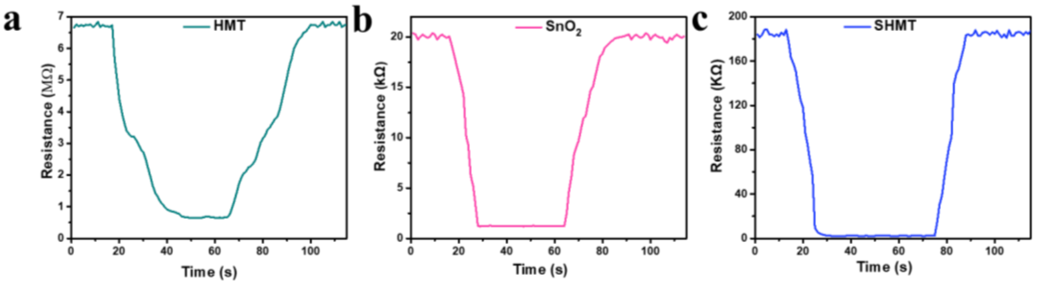

Figure S17. Dynamic changes of the electrical resistance in response to 100 ppm ethanol at their optimal temperature (350 °C, 300 °C and 350 °C, respectively) for HMT SnO_2 NCs and SHMT.

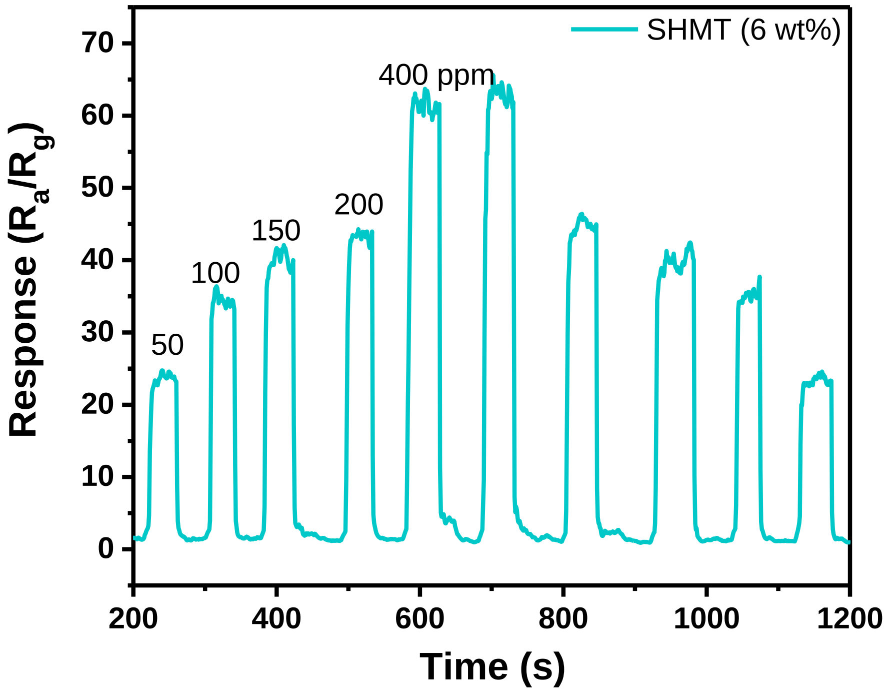

Figure S18.Response-recovery curve of SHMT (6 wt%) to low concentration acetone.

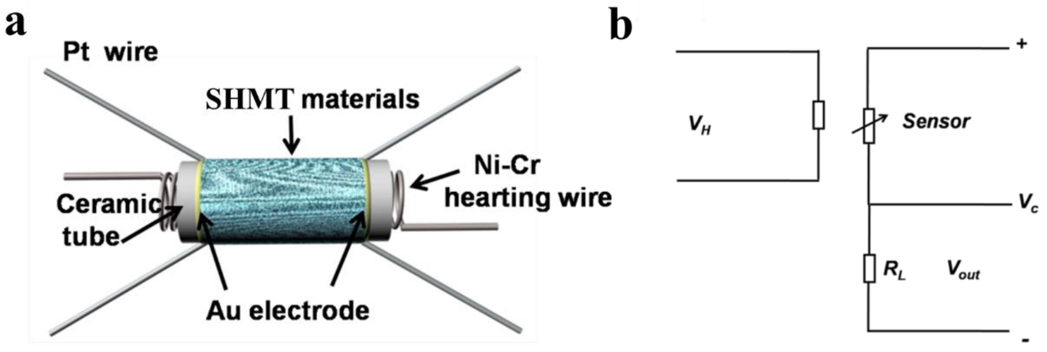

Figure S19. (a) Schematic illustration of the structure for a side-heated SHMT based gas sensor. (b) The electric circuit of gas sensing measurements.

Supplementary Reference

[S1] G. Chen, S. Ji, H. Li, X. Kang, S. Chang, Y. Wang, G. Yu, J. Lu, J. Claverie, Y. Sang, H. Liu, *ACS Appl. Mater. Interfaces* **2015,** *7*, 24950

[S2] A Hazra,. B. Bhowmik, K. Dutta, P. P. Chattopadhyay, P. Bhattacharyya, *ACS Appl. Mater. Interfaces* **2015,** *7*, 9336.

[S3] M. Z. Ahmad, A. Z. Sadek, K. Latham, J. Kita, R. Moos, W. Wlodarski, *Sens. Actuators B: Chem*. **2013,** *187*, 295

[S4] S. Singh, H. Kaur, V. N. Singh, K. Jain, T. D. Senguttuvan, *Sens. Actuators B: Chem.* **2012,** *171-172*, 899.

[S5] P. Hu, G. Du, W. Zhou, J. Cui, J. Lin, H. Liu, D. Liu, J. Wang, S. Chen, *ACS Appl. Mater. Interfaces* **2010,** *2*, 3263.

[S6] J. Deng, B. Yu, Z. Lou, L. Wang, R. Wang, T. Zhang, *Sens. Actuators B: Chem.* **2013,** *184*, 21.

[S7] X. Yang, H. Li, T. Li, Z. Li, W. Wu, C. Zhou, P. Sun, F. Liu, X. Yan, Y. Gao, X. Liang, G. Lu, *Sens. Actuators B: Chem.* **2019,** *282*, 339.

[S8] J. Liu, M. Dai, T. Wang, P. Sun, X. Liang, G. Lu, K. Shimanoe, N. Yamazoe, *ACS Appl. Mater. Interfaces* **2016,** 8, 6669.

[S9] J. Liu, T. Wang, B. Wang, P. Sun, Q. Yang, X. Liang, H. Song, G. Lu, *Sens. Actuators B: Chem*. **2017,** *245*, 551.

[S10] B. B. Wang, X. X. Fu, F. Liu, S. L. Shi, J. P. Cheng, X. B. Zhang, *J. Alloys Comp.* **2014,** *587*, 82.

[S11] G. Singh, R. C. Virpal Singh, *Sens. Actuators B: Chem.* **2019,** *282*, 373.
